# Systems biology analysis of mitogen activated protein kinase inhibitor resistance in malignant melanoma

**DOI:** 10.1186/s12918-018-0554-1

**Published:** 2018-04-04

**Authors:** Helma Zecena, Daniel Tveit, Zi Wang, Ahmed Farhat, Parvita Panchal, Jing Liu, Simar J. Singh, Amandeep Sanghera, Ajay Bainiwal, Shuan Y. Teo, Frank L. Meyskens, Feng Liu-Smith, Fabian V. Filipp

**Affiliations:** 1 Systems Biology and Cancer Metabolism, Program for Quantitative Systems Biology, University of California Merced, 2500 North Lake Road, Merced, CA 95343 USA; 2 Department of Medicine, School of Medicine, Chao Family Comprehensive Cancer Center, University of California Irvine, Irvine, CA 92697 USA; 3 The State Key Laboratory of Medical Genetics and School of Life Sciences, Department of Molecular Biology, Central South University, Changsha, 410078 China; 4 Department of Epidemiology, School of Medicine, University of California, Irvine, CA 92697 USA

**Keywords:** Cancer systems biology, Precision medicine, Omics, RNA-Seq, Transcriptomics, Upstream regulator analysis, Transcription factor, Master regulator, Regulome, Non-genomic, Rewiring, Adaptation, Genetic selection, Drug resistance, Therapy resistance, Melanoma, Melanogenesis

## Abstract

**Background:**

Kinase inhibition in the mitogen activated protein kinase (MAPK) pathway is a standard therapy for cancer patients with activating BRAF mutations. However, the anti-tumorigenic effect and clinical benefit are only transient, and tumors are prone to treatment resistance and relapse. To elucidate mechanistic insights into drug resistance, we have established an in vitro cellular model of MAPK inhibitor resistance in malignant melanoma.

**Methods:**

The cellular model evolved in response to clinical dosage of the BRAF inhibitor, vemurafenib, PLX4032. We conducted transcriptomic expression profiling using RNA-Seq and RT-qPCR arrays. Pathways of melanogenesis, MAPK signaling, cell cycle, and metabolism were significantly enriched among the set of differentially expressed genes of vemurafenib-resistant cells vs control. The underlying mechanism of treatment resistance and pathway rewiring was uncovered to be based on non-genomic adaptation and validated in two distinct melanoma models, SK-MEL-28 and A375. Both cell lines have activating BRAF mutations and display metastatic potential.

**Results:**

Downregulation of dual specific phosphatases, tumor suppressors, and negative MAPK regulators reengages mitogenic signaling. Upregulation of growth factors, cytokines, and cognate receptors triggers signaling pathways circumventing BRAF blockage. Further, changes in amino acid and one-carbon metabolism support cellular proliferation despite MAPK inhibitor treatment. In addition, treatment-resistant cells upregulate pigmentation and melanogenesis, pathways which partially overlap with MAPK signaling. Upstream regulator analysis discovered significant perturbation in oncogenic forkhead box and hypoxia inducible factor family transcription factors.

**Conclusions:**

The established cellular models offer mechanistic insight into cellular changes and therapeutic targets under inhibitor resistance in malignant melanoma. At a systems biology level, the MAPK pathway undergoes major rewiring while acquiring inhibitor resistance. The outcome of this transcriptional plasticity is selection for a set of transcriptional master regulators, which circumvent upstream targeted kinases and provide alternative routes of mitogenic activation. A fine-woven network of redundant signals maintains similar effector genes allowing for tumor cell survival and malignant progression in therapy-resistant cancer.

**Electronic supplementary material:**

The online version of this article (10.1186/s12918-018-0554-1) contains supplementary material, which is available to authorized users.

## Background

### Therapy resistance in cancer

Cancer drug resistance is a major obstacle in achieving durable clinical responses with targeted therapies. This highlights a need to elucidate the underlying mechanisms responsible for resistance and identify strategies to overcome this challenge. In malignant melanoma, activating point-mutations in the mitogen activated protein kinase (MAPK) pathway in BRAF kinase (B-Raf proto-oncogene, serine/threonine kinase, Gene ID: 673) [1–3] made it possible to develop potent kinase inhibitors matched to genotyped kinase mutations in precision medicine approaches [4–6]. In tumors expressing the oncoprotein BRAF(V600E), the inhibitor molecules vemurafenib, dabrafenib, and encorafenib are designed to lock the ATP binding site into an inactive conformation of the kinase [4], the preferred state of wild-type RAF proteins. Trametinib and cobimetinib target MAP2K7 (MEK, mitogen-activated protein kinase kinase 7, Gene ID: 5609), the BRAF target and downstream effector molecule. In MAPK signaling, combinations of specific inhibitors have proven to be superior to single-agent regimens: BRAF inhibitors (BRAFi) in combination with MEK inhibitors (MEKi) improved survival compared to single MAPK inhibitors (MAPKi) [7–10]. However, many patients responding to small molecule inhibition of the MAPK pathway will develop resistance. Ultimately, disease progression will take place and patients relapse with lethal drug-resistant disease.

### Mechanism of resistance beyond mutations

Acquired resistance has been shown to involve a diverse spectrum of oncogenic mutations in the MAPK pathway [11–15]. In addition, non-genomic activation of parallel signaling pathways has been noted [16]. Cell-to-cell variability in BRAF(V600E) melanomas generates drug-tolerant subpopulations. Selection of genetically distinct, fully drug-resistant clones arise within a set of heterogeneous tumor cells surviving the initial phases of therapy due to drug adaptation [17]. Non-genomic drug adaptation can be accomplished reproducibly in cultured cells, and combination therapies that block adaptive mechanisms in vitro have shown promise in improving rates and durability of response [18]. Thus, better understanding of mechanisms involved in drug adaptation is likely to improve the effectiveness of melanoma therapy by delaying or controlling acquired resistance.

## Methods

### Cellular models of malignant melanoma

SK-MEL-28 and A375 are human skin malignant melanoma cell lines with BRAF(V600E) activation that are tumorigenic in xenografts [19–22] (HTB-72 and CRL-1619, American Type Culture Collection, Manassas, VA). The cell lines are maintained in DMEM medium supplemented with 10% fetal bovine serum and antibiotics (10–017-CV, 35–010-CV, 30–002-CI Corning, Corning, NY). All experimental protocols were approved by the Institutional Review Boards at the University of California Merced and Irvine. The study was carried out as part of IRB UCM13–0025 of the University of California Merced and as part of dbGap ID 5094 on somatic mutations in cancer and conducted in accordance with the Helsinki Declaration of 1975.

BRAFi-resistant (BRAFi-R) models were obtained by challenging cancer cell lines with incrementally increasing vemurafenib (PLX4032, PubChem CID: 42611257, Selleckchem, Houston, TX) concentrations in the culture media. Starting at 0.25 μM, which matched the naïve half maximal inhibitory concentration (IC50) of the parental cell lines, the vemurafenib concentrations were increased every 7 days in an exponential series up to 100-fold the naïve IC50 concentrations. Following this 6-week selection protocol, vemurafenib-adapted, cancer therapy resistance models were maintained in media supplemented with 5.0 μM vemurafenib.

### Transcriptomic profiling and differential gene expression analysis

Total RNA from malignant melanoma cells was extracted using a mammalian RNA mini preparation kit (RTN10-1KT, GenElute, Sigma EMD Millipore, Darmstadt, Germany) and then digested with deoxyribonuclease I (AMPD1-1KT, Sigma EMD Millipore, Darmstadt, Germany). Complementary DNA (cDNA) was synthesized using random hexamers (cDNA SuperMix, 95,048–500, Quanta Biosciences, Beverly, MA). The purified DNA library was sequenced using a HighSeq2500 (Illumina, San Diego, CA) at the University of California Irvine Genomics High-Throughput Facility. Purity and integrity of the nucleic acid samples were quantified using a Bioanalyzer (2100 Bioanalyzer, Agilent, Santa Clara, CA). Libraries for next generation mRNA transcriptome sequencing (RNA-Seq) analysis were generated using the TruSeq kit (Truseq RNA Library Prep Kit v2, RS-122-2001, Illumina, San Diego, CA). In brief, the workflow involves purifying the poly-A containing mRNA molecules using oligo-dT attached magnetic beads. Following purification, the mRNA is chemically fragmented into small pieces using divalent cations under elevated temperature. The cleaved RNA fragments are copied into first strand cDNA using reverse transcriptase and random primers. Second strand cDNA synthesis follows, using DNA polymerase I and RNase H. The cDNA fragments are end repaired by adenylation of the 3′ ends and ligated to barcoded adapters. The products are then purified and enriched by nine cycles of PCR to create the final cDNA library subjected to sequencing. The resulting libraries were validated by qPCR and size-quantified by a DNA high sensitivity chip (Bioanalyzer, 5067–4626, Agilent, Santa Clara, CA). Sequencing was performed using 50 base pair read length, single-end reads, and more than 10^7^ reads per sample. Raw sequence reads in the file format for sequences with quality scores (FASTQ) were mapped to human reference Genome Reference Consortium GRCh38 using Bowtie alignment with an extended Burrows-Wheeler indexing for an ultrafast memory efficient alignment within the Tuxedo suite followed by Tophat to account for splice-isoforms [23, 24]. Read counts were scaled via the median of the geometric means of fragment counts across all libraries. Transcript abundance was quantified using normalized single-end RNA-Seq reads in read counts as well as reads per kilobase million (RPKM). Since single-end reads were acquired in the sequencing protocol, quantification of reads or fragments yielded similar results. Statistical testing for differential expression was based on read counts and performed using EdgeR in the Bioconductor toolbox [25]. Differentially expressed genes were further analyzed using Ingenuity Pathways Analysis (IPA, Qiagen, Rewood City, CA), classification of transcription factors (TFClass), and gene set enrichment analysis (GSEA, Broad Institute, Cambridge, MA) [26, 27]. For real-time quantitative polymerase chain reaction (RT-qPCR) validation of RNA-Seq signals of differentially expressed target genes in BRAFi-R melanoma cells, gene expression profiles were analyzed using the ΔΔ threshold cycle (CT) method. Oligonucleotides spanning exon-exon-junctions of transcripts were used for RT-qPCR validation (Additional file 1: Table 1). Triple replicate samples were subjected to SYBR green (SYBR green master mix, PerfeCTa® SYBR® Green FastMix®, 95072-05k, Quanta Biosciences, Beverly, MA) RT-qPCR analysis in an Eco system (Illumina, San Diego). CT values were normalized using multiple housekeeping genes like actin beta (ACTB, Gene ID: 60), cyclophilin A (PPIA, peptidylprolyl isomerase A, Gene ID: 5478) and RNA polymerase II subunit A (POLR2A, GeneID: 5430).

### Inhibitor cytotoxicity studies

Chemical BRAFi against BRAF(V600E), vemurafenib, was dissolved in dimethyl sulfoxide (DMSO, Sigma) as a 10.0 mM stock solution and used in treatments in final concentrations between 0.01 μM and 50.0 μM. Melanoma control experiments were carried out in the presence of equivalent amounts of DMSO solvent without drug. Cell viability was determined using a 2,3-bis(2-methoxy-4-nitro-5-sulfophenyl)-2H-tetrazolium-5-carboxanilide (XTT, X4626, Sigma EMD Millipore, Darmstadt, Germany) absorbance assay by subtracting background readout at 650 nm from response readout at 570 nm wavelength. IC50 concentrations were determined after 72 h of drug treatment between 0.01–100 μM in two-fold dilution series. Analysis was performed using CalcuSyn (v2.0, Biosoft, Cambridge, UK).

### Melanin quantification

Melanin pigment production of cultured cells was determined by colorimetric measurements normalized for total protein levels in arbitrary units [28, 29]. Melanoma cells were harvested by centrifugation at 3000 rpm (3830 g, Z326K, Labnet International, Edison, NJ) and dissolved in either 1.0 N NaOH for melanin assay or lysis 250 for protein assay. The cell lysates were sonicated, incubated at room temperature for 24 h, and cleared by centrifugation at 13,000 rpm for 10 min (17,000 g, Z326K, Labnet International, Edison, NJ). The absorption of the supernatant was measured at 475 nm in a spectrophotometer (Smartspec3000, Bio-Rad, Carlsbad, CA). Cells were lysed in mild denaturing conditions in lysis 250 buffer (25 mM Tris, [pH 7.5], 5 mM EDTA, 0.1% NP-40, 250 mM NaCl) containing proteinase inhibitors (10 μg/ml aprotinin, 10 μg/ml leupeptin, 10 μg/ml pepstatin, 5 μg/ml antipain, 1 mM phenylmethylsulfonyl fluoride). The total protein amount in the lysates was quantified using a colorimetric Bradford assay (5000001, Bio-Rad, Richmond, CA) at 595 nm and an incubation time of 30 min [30].

## Results

### Generation of BRAFi-resistant melanoma cell lines

**Fig. 1:**
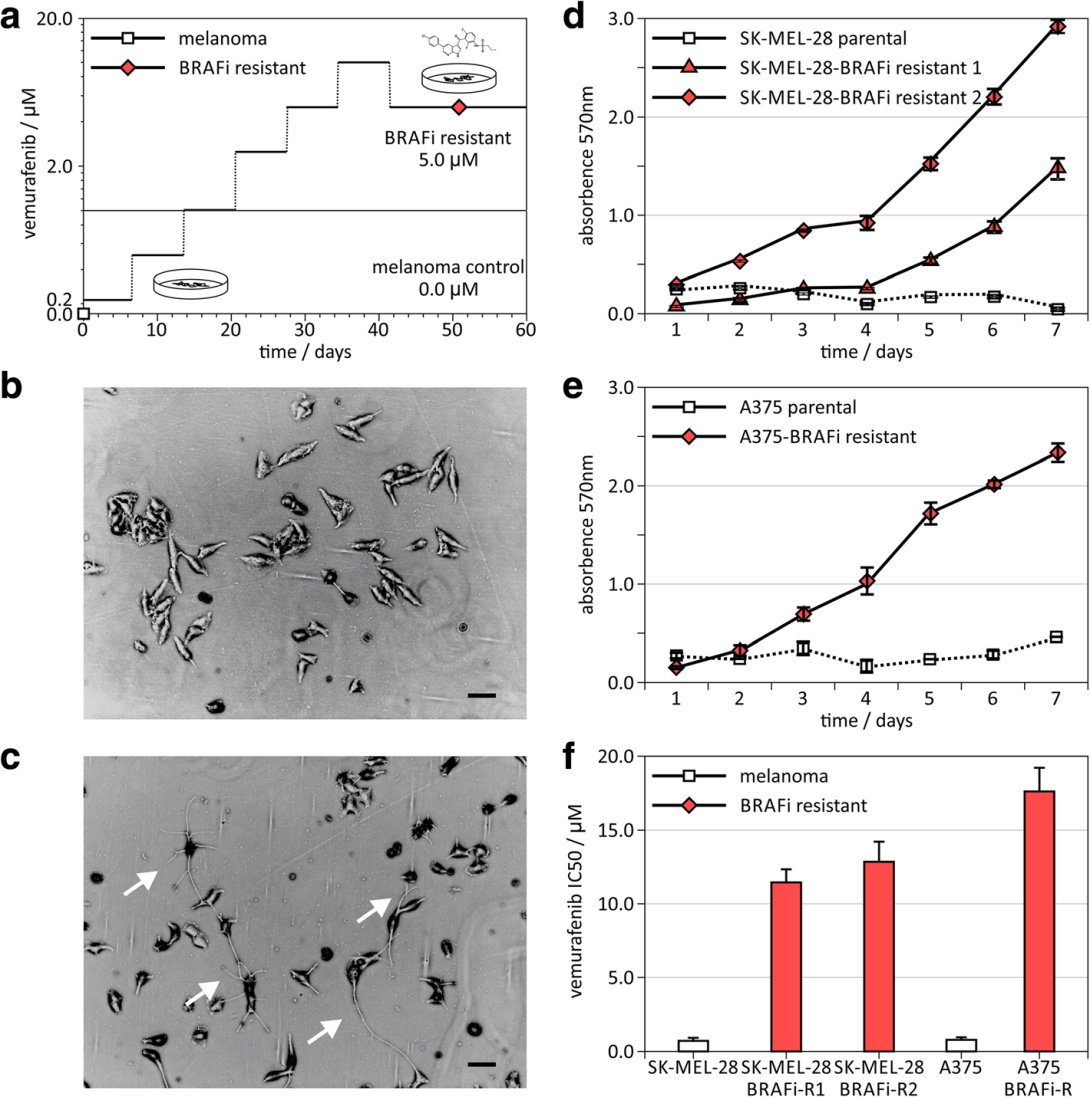
Establishing mitogen activated protein kinase inhibitor-resistant melanoma models. **a** A mitogen activated protein kinase BRAF inhibitor-resistant (BRAFi-R) model was established using SK-MEL-28 and A375 malignant melanoma cell lines. Schedule of administered concentrations of mitogen activated kinase inhibitor, vemurafenib. **b** Phase contrast images of control SK-MEL-28 parental melanoma cell lines and (**c**) BRAF inhibitor-resistant SK-MEL-28-BRAFi-R melanoma cell line 1. Black bar indicates 1.0 μm. White arrows in image of resistant cell lines point to cellular structures typical for differentiated melanocytes. **d** and **e** Cell viability assay on melanoma cell lines at 10 μM vemurafenib. Absorption in XTT assay is measured at 570 nm. White squares indicate control melanoma cell lines, red triangles and diamonds show melanoma BRAFi-R model. **f** IC50 concentrations of vemurafenib of control and drug-resistant cancer cell lines

The parental melanoma cell lines SK-MEL-28 and A375 were exposed to incrementally increasing concentrations of the mutant-BRAF inhibitor vemurafenib (Fig. 1a). At the initial inhibitor concentration matching the IC50 of vemurafenib in the naïve parental melanoma cells [11, 31] cell proliferation decreased. Surviving cells were propagated and subjected to an exponential series of increasing vemurafenib concentrations until BRAFi-R sublines were obtained tolerating at least 5 μM vemurafenib in the culture media with similar cell proliferation rates as the parental cell lines of 0.67 doublings per day.

Some BRAFi-R cell lines showed structures typically observed in differentiated melanocytes (Fig. 1b-c). In the presence of 5 μM vemurafenib, however, the parental cells were not able to grow but the resistant cells proliferated comparable to naïve cell lines (Fig. 1d-e). For the SK-MEL-28 cell line, two resistant sublines were established. The resistant sublines displayed IC50 values of 11.5 ± 0.9 μM and 13.3 ± 1.2 μM for SK-MEL-28-BRAFi-R1 and SK-MEL-28-BRAFi-R2 respectively, which is approximately 10–20 fold of the IC50 in a low micro-molar range for the parental cells with 0.74 ± 0.05 μM. For the A375 cell line, the IC50 of the A375-BRAFi-R cell line was observed at 17.7 ± 1.5 μM, 22.7 fold of IC50 for the parental A375 cells with 0.78 ± 0.22 μM (Fig. 1f).

### Transcriptomic profiling identifies non-genomic rewiring of treatment-resistant cancer cells

**Fig. 2:**
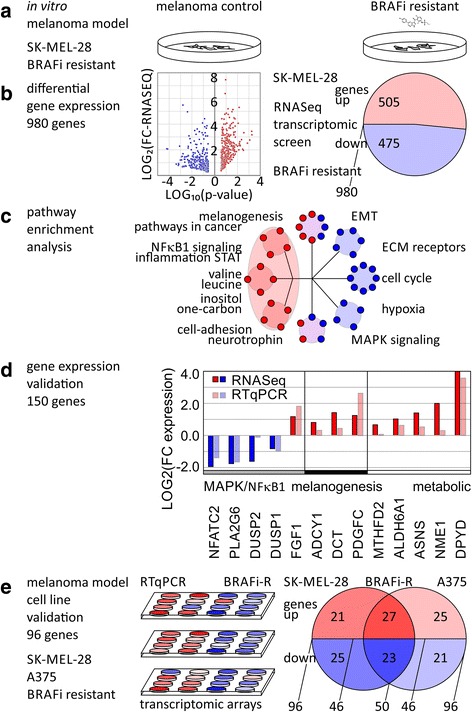
Transcriptomic profiling of BRAF inhibitor resistance in cellular models of malignant melanoma. **a** Establishing cellular models of mitogen activated protein kinase inhibitor resistance using SK-MEL-28 malignant melanoma cell line and the BRAF inhibitor, vemurafenib. **b** Transcriptomics RNA-Seq analysis identifies 980 differentially expressed genes between BRAF inhibitor-resistant (BRAFi-R) cellular model vs control. **c** Enrichment analysis of up- and downregulated gene sets shows shift in metabolic and signaling pathways. **d** Validation by transcriptomic profiling of identified genes by RT-qPCR. **e** Comparison and validation of resistance model using melanoma cell lines SK-MEL-28 and A375 by transcriptomics RT-qPCR arrays

We conducted transcriptomic gene expression profiling of BRAFi treatment-resistant SK-MEL-28-BRAFi-R1 and SK-MEL-28-BRAFi-R2 cell lines by RNA-Seq and looked for differential expression versus the parental SK-MEL-28 cell line. In total, 980 unique transcripts showed significant differential expression in RNA-Seq experiments with *p* values below 0.05, absolute log-fold change (LOG(FC)) greater or equal 1.0 (Fig. 2a-b). The differentially expressed genes included 505 upregulated transcripts and 475 downregulated transcripts (Additional file 1: Table S2–3). We subjected the identified directional sets to pathway enrichment analysis (Additional file 1: Table S4). Distinct clusters stood out and showed significant enrichment with *p* values below 0.05 and *q* values below 0.10 (Fig. 2c). Melanogenesis and pathways in cancer, inflammation, nuclear factor kappa-light-chain-enhancer of activated B cells (NFκB) and signal transducer and activator of transcription (STAT) signaling, metabolic pathways including alanine, tyrosine, valine, leucine, inositol, one-carbon metabolism, cell-adhesion molecules, neurotrophin signaling were over-represented in the upregulated dataset. MAPK signaling and epithelial-mesenchymal transition (EMT) were differentially expressed and characterized by both strong up- and downregulation. Extra-cellular matrix (ECM) receptors, cell cycle, and hypoxia signaling were enriched in the downregulated dataset. Of the 980 differential expressed genes, we validated expression changes of 150 genes by RT-qPCR (Fig. 2d, Additional file 1: Table S3). Of these, a majority, 64.0% (96 of 150), responded significantly (with *p* values below 0.05) in the same direction as RNA-Seq data for treatment-resistant melanoma. When both treatment resistance models of SK-MEL-28 and A375 were taken into consideration, about half of the tested genes, 50 of 96, showed consistent regulation (Fig. 2e, Additional file 1: Table S3). Genes in MAPK signaling included nuclear factor of activated T-cells 2 (NFATC2, Gene ID: 4773), phospholipase A2 group VI (PLA2G6, Gene ID: 8398), dual specificity phosphatase 1 (DUSP1, Gene ID: 1843), and dual specificity phosphatase 2 (DUSP2, Gene ID: 1844), which were downregulated in the BRAFi-R cells compared to control. Genes contributing to melanogenesis adenylate cyclase 1 (ADCY1, Gene ID: 107), dopachrome tautomerase (DCT, TYRP2, Gene ID: 1638), and platelet derived growth factor C (PDGFC, Gene ID: 56034) were upregulated. Lastly, metabolic regulators such as methylenetetrahydrofolate dehydrogenase 2 (MTHFD2, Gene ID: 10797) for folate metabolism, asparagine synthetase (ASNS, Gene ID: 440) for amino acid metabolism, and NME/NM23 nucleoside diphosphate kinase 1 (NME1, Gene ID: 4830) and dihydropyrimidine dehydrogenase (DPYD, Gene ID: 1806) for pyrimidine metabolism were significantly upregulated (Fig. 2d). Taken together, the adaptive transcriptomic changes were validated in two distinct melanoma models, SK-MEL-28 and A375, both cell lines with metastatic potential showed differential expression of MAPK signaling while activating alternative mitogenic signaling interactions and metabolic processes.

### Upstream regulator analysis suggests control by transcription factor families

**Fig. 3:**
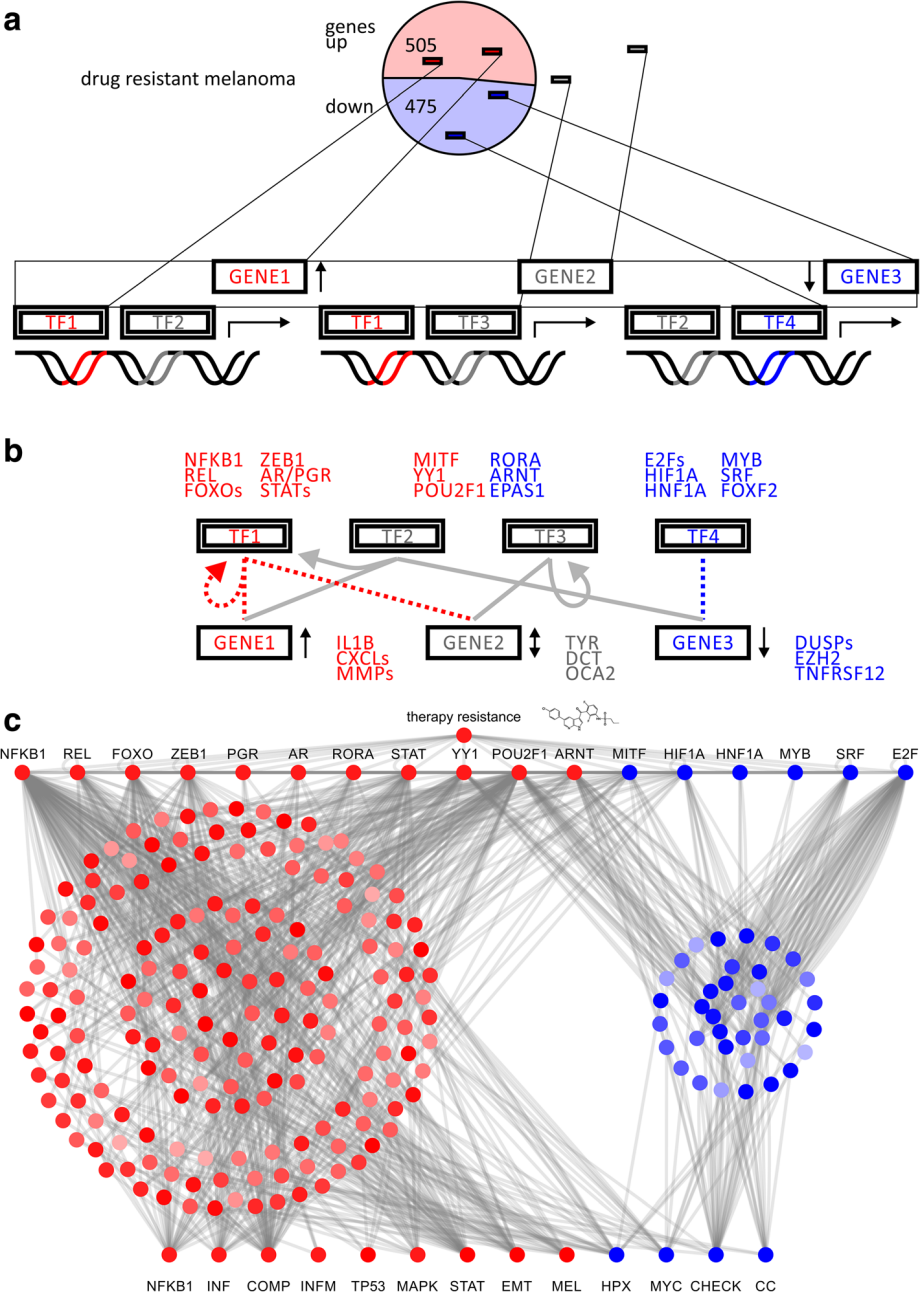
Transcription factor motif analysis of mitogen activated protein kinase inhibitor resistance in cellular models of malignant melanoma. **a** Schematic representation of differentially expressed genes in drug resistance model and transcription factor motifs associated with regulated target genes. Upregulated and downregulated factors are depicted in red and blue, respectively. **b** Hierarchical transcription factor network with master regulators on top and downstream targets at bottom. Sets of transcription factor target genes are identified in enrichment analysis based on sequence motifs. **c** Hierarchical network model illustrates how therapy resistance in cancer selects for specific transcriptional master regulators to rewire target genes in effector pathways in a concerted fashion

Next, the gene list was subjected to hierarchical transcription factor motif analysis to identify master regulators [32]. We asked whether any of the enriched transcription factor motif families were represented in the differential gene expression data. In detail, we looked for transcription factors as well as their target genes whose promoters show respective transcription factor binding sites among the same list of regulated genes (Fig. 3a). It is expected that differentially expressed transcription factors show motif enrichment in promoter sites of significantly deregulated target genes. Further, identified target genes with enriched transcription factor motifs will have major contributions to significantly deregulated pathways under treatment resistance (Fig. 3b). A network illustration of transcriptional master regulators, target genes, and dysregulated effector network upon treatment resistance demonstrates transcriptional synergy (Fig. 3c). Upregulated transcription factor families included Rel homology region (RHR) NFκB-related factors, forkhead box (FOX), Zinc finger E-box-binding homeobox domain factors (ZEB), nuclear steroid hormone receptor subfamily 3 (NR3C, androgen receptor and progesterone receptor), hypoxia-inducible and endothelial PAS domain-containing factors (HIF, EPAS), and the cell cycle transcription factor family (E2F) (Fig. 3b). Downstream enriched target genes comprised members of interleukin (IL), chemokine receptor (CXCL), matrix metallo proteinase (MMP) families, transcription factors forkhead box O1 (FOXO1, Gene ID: 2308), endothelial PAS domain protein 1 (EPAS1, HIF2A, Gene ID: 2034) and melanogenesis associated metabolic genes, tyrosinase (TYR, OCA1, Gene ID: 7299), DCT, and melanosomal transmembrane protein (OCA2, oculocutaneous albinism II, Gene ID: 4948). Downregulated transcription factors included forkhead box F2 (FOXF2, Gene ID: 2295), which has DUSP2 or transforming growth factor beta 3 (TGFB3, Gene ID: 7043) as target genes. Upstream regulator analysis suggested gene expression changes of nuclear factor kappa B subunit 1 (NFKB1, Gene ID: 4790, V$NFKB_Q6, motif M11921) in complex with REL proto-oncogene (REL Gene ID: 5966, V$CREL_01, motif M10143), EMT modulator zinc finger E-box binding homeobox 1 (ZEB1, Gene ID: 6935, V$AREB6_01, M11244), forkhead box (V$FOXO1_01, motif M11512), and hypoxia inducible factor family transcription factors (V$HIF1_Q3, motif M14011) as master regulators of transcriptional effector networks upon BRAFi treatment resistance.

### Validation of pathway rewiring in drug resistance in multiple cell lines by transcriptomics arrays

**Fig. 4:**
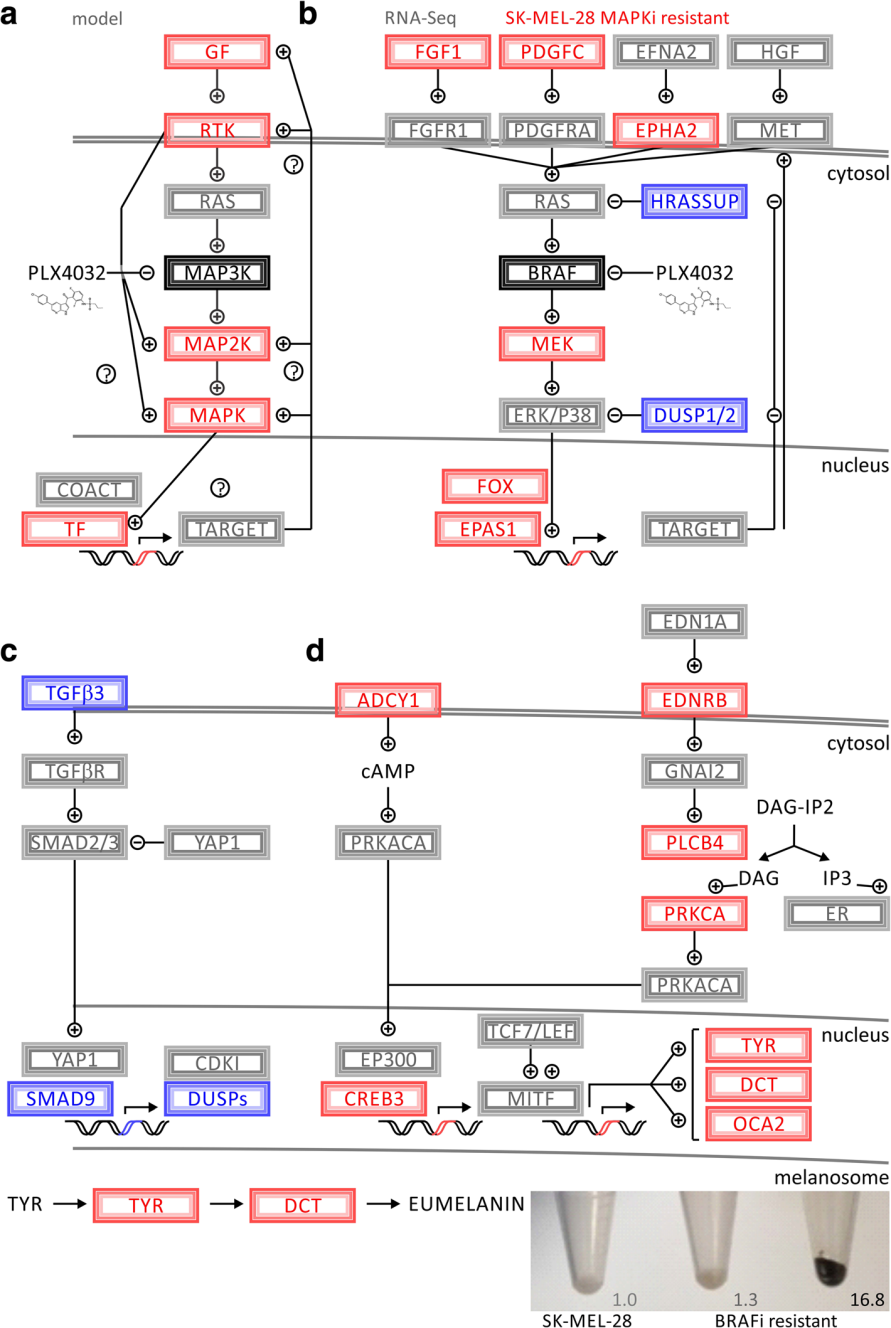
Pathway analysis of BRAF kinase inhibitor resistance shows alternative activation of MAPK targets and pigmentation. **a** Schematic representation of regulatory network involving drug inhibition and non-genomic selection for differential expression of driver genes that can circumvent suppressed signaling. **b** Deregulation of MAPK signaling with RNA-Seq data is mapped in red and blue for differential upregulation and downregulation, respectively. **c** Modulation of TGFB signaling leads to downregulation of dual specific phosphatases, which are required to switch MAPK signaling off. **d** Interconnectedness between G-protein signaling and melanogenesis. Alternative activation of melanoma pathways leads to increased eumelanin synthesis and mitogenic survival. Photograph of cell pellets of melanoma cell models and detected melanin. Left shows SK-MEL-28 melanoma cell line, middle and right shows two different SK-MEL-28-BRAFi-resistant melanoma cell lines with elevated melanin production

Transcriptome analysis of reversible drug resistance identified distinct pathways that allowed for circumvention of BRAF blockage (Fig. 4a). Cell-to-cell variability in combination with drug exposure selects for distinct sub-populations of MAPKi-resistant (MAPKi-R) cell lines. In a hierarchical fashion, transcriptional master regulators promote a distinct set of target genes resulting in circumvention of MAPK inhibition. Receptor activation by fibroblast growth factor 1 (FGF1, Gene ID: 2246) or PDGFC can lead to activated receptor tyrosine signaling parallel to canonical MAPK signaling [16] (Fig. 4b). In addition, downregulation of tumor suppressors reengages mitogenic signaling. The dual specific phosphatases, DUSP1 and DUSP2, have the ability to switch MAPK signaling off and rank among the top downregulated hits. Thus, downregulation of dual specific phosphatases facilitates and reinforces alternative MAPK effector activation under BRAF blockage (Fig. 4b). One of the mitogen-activated protein kinase 1 (MAPK1, ERK2, Gene ID: 5594) effector targets, transcription factor EPAS1, showed upregulation and the ability to maintain its transcriptional program. The pro-apoptotic program of TGFB3 was downregulated including SMAD family member 9 (SMAD9, Gene ID: 4093) and DUSP1/2 (Fig. 4c). Adenylate cyclase, G-protein, and phospholipase signaling are alternative cascades observed in cutaneous and uveal melanoma (Fig. 4d). Upregulation of ADCY1, endothelin receptor type B (EDNRB, Gene ID: 1910), phospholipase C beta 4 (PLCB4, Gene ID: 5332), and cAMP responsive element binding protein 3 (CREB3, Gene ID: 10488) promote MITF activity, the master transcription factor for pigmentation genes. Downstream metabolic enzymes, TYR and DCT, are both MITF target genes and contribute to enhanced eumelanin production observed in some therapy-resistant cell lines. The observed pigmentation showed a wide range of from 1.3-fold to up to 16.8-fold upregulation (Fig. 4d). While both cell lines showed dysregulation of melanogenesis, the regulators and effectors involved were different. SK-MEL-28-BRAFi-R2 has ASIP prominently expressed (TYR (2.1), DCT (2.8), tyrosinase related protein 1 (TYRP1, OCA3, Gene ID: 7306) (0.5), MITF (0.7), agouti signaling protein (ASIP, Gene ID: 434) (18.9)), while A375-BRAFi-R showed strongest regulation of TYRP1 and MITF (TYR (0.34), DCT (0.24), TYRP1 (41.8), MITF (2.94), ASIP (0.41)).

In summary, upregulation of growth factors or receptors triggers signaling pathways circumventing BRAF blockage. Changes in amino acid and one-carbon metabolism support cellular proliferation despite inhibitor treatment. In addition, alternative MAPK signaling coincides with differential response of melanogenesis and pigmentation pathways, which partially overlap with MAPK effectors. In particular, NFKB1, REL, ZEB1, FOXO1, and EPAS1 may serve as master regulators to enact broad transcriptional changes implemented in altered cascades of MAPK, TGFB, ADCY, and MITF signaling.

## Discussion

Activation of the MAPK pathway is the central and most common oncogenic event in the pathogenesis of malignant melanoma [3, 33]. About 50% of all melanoma patients have activating somatic mutations in the activator loop involving L597, T599, V600, and K601 switching proto-oncogene BRAF into a constitutively active protein kinase and cancer driver. Such activation is supported by somatic copy number amplifications of chromosome 7 [34], often coinciding with somatic V600E/G/K/M/R mutations. Another 20–30% of the patients show non-genomic activation of BRAF by transcriptional upregulation or post-translational modification induced by somatic mutations of upstream signaling molecules like KIT proto-oncogene receptor tyrosine kinase (KIT, Gene ID: 3815), proto-oncogene neuroblastoma RAS viral oncogene homolog (NRAS, Gene ID: 4893), or loss-of-function neurofibromin 1 (NF1, Gene ID: 4763). Constitutively activated BRAF phosphorylates MAPK1 and downstream kinases resulting in mitogenic signaling, proliferation, and cell growth. Integrated into this cellular program is negative feedback resulting in reduction of NRAS expression [35, 36].

### Genomic and non-genomic mechanisms of therapy resistance

Genomic sequencing has facilitated the understanding of acquired resistance mechanisms to MAPKis [14–16, 37–40]. Detected genetic aberrations included mutations in NRAS, MAPK1/2, phosphatidylinositol-4,5-bisphosphate 3-kinase catalytic subunit alpha (PIK3CA, Gene ID: 5290), and phosphatase and tensin homolog (PTEN, Gene ID: 5728). Somatic melanoma mutations provide examples of how single, well-defined genomic events can confer resistance against vemurafenib treatment. In contrast, transcriptomic as well as epigenomic regulation can provide insight into resistance states that may involve larger networks. Eventually, resistance-conferring genomic, epigenomic, and transcriptomic alterations result in sustained mitogenic effector signaling and persist to promote proliferation.

### Network rewiring of therapy-resistant melanoma

The transcriptomic profiles revealed a network of genes involved in adenylate cyclase signaling conferring resistance and contributing to melanogenesis. ADCY1 and CREB3 are prominent members of the melanogenesis pathway exhibiting mitogenic control and MITF activation. Similarly, a gain-of-function screen confirmed a cyclic-AMP-dependent melanocytic signaling network including G-protein-coupled receptors, adenylate cyclase, protein kinase cAMP-activated catalytic subunit alpha (PRKACA, Gene ID: 5566), and cAMP responsive element binding protein 1 (CREB1, Gene ID: 1385) [41]. The MAPK pathway negatively regulates MITF protein level as well as activity [29], which in turn regulates a series of cell cycle regulating genes. In particular, P16INK4A and P21CIP1, gene products of cyclin dependent kinase inhibitor 2A (CDKN2A, Gene ID: 1029) and cyclin dependent kinase inhibitor 1A (CDKN1A, Gene ID: 1026), respectively, differentiation genes TYR, DCT, TYRP1 as well as survival genes B-cell lymphoma 2 apoptosis regulator (BCL2, Gene ID: 596) and BCL2 family apoptosis regulator (MCL1, Gene ID: 4170) are effector genes under the control of MITF. Indeed, inhibition of MITF increases sensitivity to chemotherapy drugs [42]. In contrast, upregulation of MITF in therapy-resistance may present itself as a survival mechanism, which coincides with upregulation of melanin, hence it may serve as prognostic biomarker for drug adaptation.

Dual specific phosphatases (DUSPs) act downstream of BRAF on phosphorylated MAPK members to provide attenuation of signal. Loss of DUSP activity results in constitutive activation of the pathway. Prominent members of this family DUSP1 and DUSP2 are consistently downregulated at the transcriptional level. In prior clinical studies, somatic mutation of DUSP4 in MAPKi-R has been reported [39]. Although in that case a genomic mechanism of resistance was utilized, the outcome of reduced DUSP activity by genomic or transcriptomic changes is equivalent and leads to persistent triggering of MAPK effectors.

### Metabolic support of therapy resistance

Metabolic genes support the rewiring of acquired resistance and have been shown to play an intricate role in the malignancy of skin cutaneous tissues. Glutamine and glucose metabolism showed sensitivity to combinations of MAPKi and metabolic inhibitors in preclinical studies [43]. The transciptomic profiles identified key enzymes in related, branching glycolytic pathways of serine, folate and pyrimidine metabolism. A cancer systems biology analysis of skin cutaneous melanoma brought forward a new master regulator and diagnostic target in cancer metabolism. Somatic mutations of DPYD have the ability to reconfigure and activate pyrimidine metabolism promoting rapid cellular proliferation and metastatic progression [44].

### Concertation of transcriptional regulators

The forkhead box family of transcription factors is an important downstream target of the MAPK pathway and is currently being considered as a new therapeutic target in cancer, including melanoma therapy [45]. In epithelial cells, these transcriptional factors are directly involved in the expression of cyclin dependent kinase inhibitors and CDKN2A gene under the control of TGFβ [46, 47]. Both downregulation of anti-apoptotic targets as well as activation of proliferative metabolism have been observed as mechanisms contributing to MAPKi-R. Downregulation of FOXF2 has been shown to promote cancer progression, EMT, and metastatic invasion [48]. In contrast, a different member of the FOX family, the stem cell transcription factor forkhead box D3 (FOXD3) has been identified as an adaptive mediator of the response to MAPK pathway inhibition selectively in mutant BRAF melanomas [49, 50].

We have discovered non-genomic rewiring of pathways in chemotherapy resistance by RNA-Seq data and validated gene targets in two cell lines by transcriptomics arrays. Perturbation of these resistance pathways by drug molecules, RNA interference, or genomic editing will corroborate the translational impact of identified gene targets. The established cell culture models of treatment resistance provide a broadly applicable platform to utilize high-throughput screening tools in the search for effective combinations of targeted therapies in cancer.

## Conclusion

The MAPK pathway undergoes major rewiring at the transcriptional level while acquiring inhibitor resistance. The outcome of such transcriptional plasticity is dysregulation at the level of different upstream master regulators, while maintaining similar effector genes. Combination therapies including targeted approaches and immune checkpoint inhibition are promising and rapidly improving. For these therapies to show durable, progression-free success in the clinical setting, adaptation mechanisms of treatment resistance need to be understood. Cellular model systems in combination with transcriptome-wide analyses provide insight into how non-genomic drug adaptation is accomplished. Ongoing efforts are focused on utilizing the established preclinical models to overcome drug adaptation as well as precision medicine profiling of cancer patients. Over time, a better understanding of mechanisms involved in drug adaptation is likely to improve the effectiveness of melanoma therapy by delaying or controlling acquired resistance.

## Additional file

Additional file 1: Table S1-S4 are compiled as supplementary information. Table S1: Oligonucleotides for RT-qPCR arrays. Table S2: Differentially expressed gene set based on RNA-Seq data. Table S3: Validated transcripts. Table S4: enrichment based on directional transcriptomic data. (XLSX 94 kb)

## Abbreviations

BRAF: B-Raf proto-oncogene, serine/threonine kinase
BRAFi: BRAF inhibitor
BRAFi-R: BRAFi-resistant
cDNA: complementary DNA
CT: threshold cycle
DMSO: dimethyl sulfoxide
DUSPs: dual specific phosphatase
ECM: extra-cellular matrix
EMT: epithelial-mesenchymal transition
FOX: forkhead box
HIF: hypoxia-inducible factor
IC50: half maximal inhibitory concentration
LOG(FC): log-fold change
MAPK: mitogen activated protein kinase
MAPKi: MAPK inhibitor
MAPKi-R: MAPKi-resistant
MEKi: MEK inhibitor
XTT: 2,3-bis(2-methoxy-4-nitro-5-sulfophenyl)-2H-tetrazolium-5-carboxanilide
RNA-Seq: next generation mRNA transcriptome sequencing
RPKM: RNA-Seq single-end reads in reads per kilobase million
RT-qPCR: real-time quantitative polymerase chain reaction
STAT: signal transducer and activator of transcription
